# Peak Detection Method Evaluation for Ion Mobility Spectrometry by Using Machine Learning Approaches 

**DOI:** 10.3390/metabo3020277

**Published:** 2013-04-16

**Authors:** Anne-Christin Hauschild, Dominik Kopczynski, Marianna D’Addario, Jörg Ingo Baumbach, Sven Rahmann, Jan Baumbach

**Affiliations:** 1 Computational Systems Biology Group, Max Planck Institute for Informatics, Saarbrücken, Germany; E-Mail: jan.baumbach@imada.sdu.dk; 2 Cluster of Excellence for Multimodal Computing and Interaction. Saarland University, Saarbrücken, Germany; 3 Computer Science XI and Collaborative Research Center SFB 876, TU Dortmund, Germany; E-Mails: dominik.kopczynski@tu-dortmund.de (D.K.); marianna.daddario@uni-dortmund.de (M.D.); sven.rahmann@tu-dortmund.de (S.R.); 4 B & S Analytik, BioMedizinZentrum Dortmund, Germany; E-Mail: baumbach@bs-analytik.de; 5 Genome Informatics, Human Genetics, Faculty of Medicine, University of Duisburg-Essen, Essen, Germany; 6 Computational Biology Group, Department of Mathematics and Computer Science, University of Southern Denmark, Odense, Denmark

**Keywords:** mcc/ims, peak detection, machine learning, ion mobility spectrometry, spectrum analysis

## Abstract

Ion mobility spectrometry with pre-separation by multi-capillary columns (MCC/IMS) has become an established inexpensive, non-invasive bioanalytics technology for detecting volatile organic compounds (VOCs) with various metabolomics applications in medical research. To pave the way for this technology towards daily usage in medical practice, different steps still have to be taken. With respect to modern biomarker research, one of the most important tasks is the automatic classification of patient-specific data sets into different groups, healthy or not, for instance. Although sophisticated machine learning methods exist, an inevitable preprocessing step is reliable and robust peak detection without manual intervention. In this work we evaluate four state-of-the-art approaches for automated IMS-based peak detection: local maxima search, watershed transformation with IPHEx, region-merging with VisualNow, and peak model estimation (PME). We manually generated a gold standard with the aid of a domain expert (manual) and compare the performance of the four peak calling methods with respect to two distinct criteria. We first utilize established machine learning methods and systematically study their classification performance based on the four peak detectors’ results. Second, we investigate the classification variance and robustness regarding perturbation and overfitting. Our main finding is that the power of the classification accuracy is almost equally good for all methods, the manually created gold standard as well as the four automatic peak finding methods. In addition, we note that all tools, manual and automatic, are similarly robust against perturbations. However, the classification performance is more robust against overfitting when using the PME as peak calling preprocessor. In summary, we conclude that all methods, though small differences exist, are largely reliable and enable a wide spectrum of real-world biomedical applications.

## 1. Introduction

Over the last decade the focus in ion mobility spectrometry (IMS) research has widened, now including biotechnological and medical applications, such as patient breath analysis and monitoring [1], identification of bacterial strains and fungi [2,3], cancer sub-typing, and skin volatile detection [4], just to name a few. A recently established approach to pre-separate the analytes before they enter the spectrometer is to couple an IMS with a multi-capillary column (MCC), providing the potential for detecting volatile organic compound (VOC) with a much higher resolution (see Preliminaries below). The MCC/IMS BioScout device [5,6] built by B&S Analytik (Dortmund, Germany) [7] combines these two technologies and is particularly designed for medical applications. Here, it was applied to measure the metabolic output in human breath. We utilize 69 measurements: 39 from different patients suffering from the same disease and 30 healthy “patients” in a control group. Note that the patient data is sensitive and confidential such that we cannot present more details here. Our goal in this scenario is a classifier distinguishing “healthy” from “not healthy” patients, which shall be a support for a physician by proposing the most likely patients condition. Since the daily increasing number of measurements, each with several dozens of potential peaks, exceeds the ability of a human to find a pattern within the data, the necessity of an automated data analysis and classification emerges. 

The first step in automatic high-throughput MCC/IMS data analysis is peak detection as each so-called peak represents a specific analyte in the exhaled air (see Preliminaries below). Such an automatic peak detection method should be as accurate as a human annotation, but fast enough to cope with thousands of measurements. Several peak detection algorithms for MCC/IMS data have been proposed and described in the literature [8,9,10,11]. As of yet, they are generally evaluated using intrinsic quality measures, *i.e*., criteria that can be derived from the data (such as goodness of fit of the peak models to the initially measured data). However, our final goal is not to describe a peak mathematically as exactly as possible but to optimize the classification performance that will allow us to decide whether a patient is healthy or not. Hence, we provide a different evaluation strategy that specifically aims at this criterion. We will evaluate a selection of four state-of-the-art peak detection methods according to data-independent criteria, *i.e*., criteria that are not contained within the measurement data itself but reflect their performance and robustness regarding the final task: biomedical decision making. 

**Figure 1:**
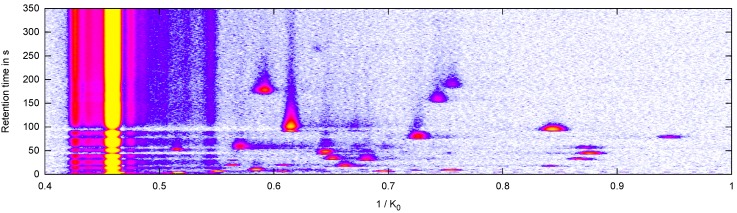
Heat map of an MCC/IMS measurement. X-axis: inverse reduced mobility 1/*K*_0_ in Vs/cm^2^; Y-axis: retention time *r* in seconds; signal: white (lowest) < blue < purple < red < yellow (highest), reaction ion peak (RIP) at 1/*K*_0_ = 0.46 Vs/cm^2 ^.

Each peak detection method transforms a raw data measurement (a matrix of values, see an example heat map representation in Figure 1) into a set of peak descriptors. These will be used as input for two sophisticated machine learning classification algorithms, a support vector machine and a random forest model. We will evaluate each peak detection method by its contribution to predict the correct binary decisions (disease present or absent). Furthermore, we evaluate the robustness of this classification process. In addition, we compare the four computational tools introduced below with a manually selected peak set by experts in the MCC/IMS data analysis field where peaks in every single measurement are marked by hand. In the remainder we call this very time-consuming “method” the gold standard. 

**Figure 2:**
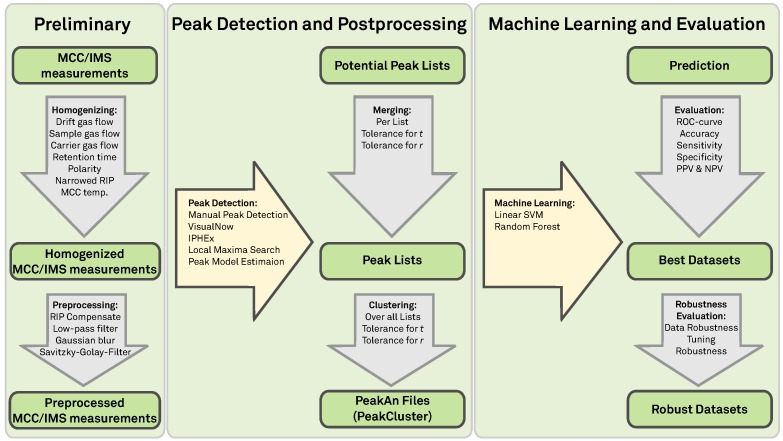
Overview: Evaluation pipeline.

Figure 2 gives an overview of the following sections and the structure of the comparison process. In Section 2 we discuss the technical background of the MCC/IMS device and pre-processing steps taken on the raw data. Section 3 gives an overview about the peak detection methods we evaluate and describes which machine learning and evaluation methods we apply to assess the quality of the peak detection methods. In Section 4 we describe our comparison results and finally discuss these findings in Section 5 and concludes the paper. 

## 2. Preliminaries

### 2.1. MCC/IMS Devices

IMS technology has been developed and improved since the 1970s. An IMS device can detect VOCs even at the concentration level of picogram per liter. We briefly present the working principles of an MCC/IMS device here. We give typical values exemplarily for the BioScout device; details are provided by Baumbach *et al.* [6]. 

**Figure 3:**
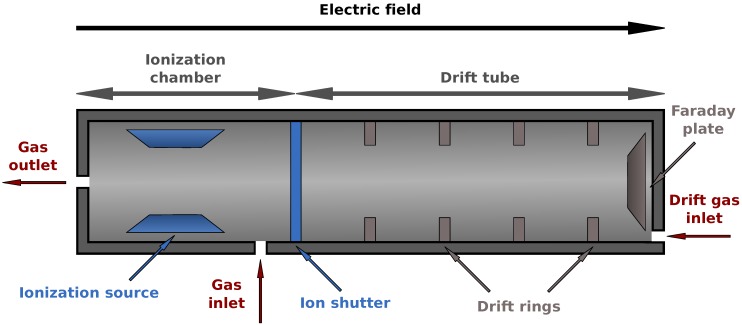
Schematic view of an IMS device. After ionization the analytes (charged molecules) are accelerated by an electric field and move towards a Faraday plate to which they transfer their charge. This is measured as a voltage signal. A drift gas flows in the opposite direction, thereby causing collisions that separate the analytes by their chemical properties. See text for details.

An ion mobility spectrometer (IMS) is divided into two parts, consider Figure 3. Analytes are first injected into the ionization chamber. Typically a radioactive source (^63^Ni) is used for ionizing the molecules afterwards though different methods are available here. After the ion shutter opens, they are pulled towards a Faraday plate by an electric field created by drift rings. From the opposite direction drift gas is injected, which de-accelerates the ions. When the ions hit the Faraday plate they transfer their charge allowing us to measure a voltage signal over time. Depending on their mass and specific shape and other chemical properties, they collide more or less frequently with the drift gas, described by the collision cross section. Thus the drift time (time of flight inside the drift tube) varies for different compounds. The whole process takes about 50 ms. 

To distinguish different compounds with similar drift time, a so-called multi-capillary column (MCC) is coupled to the IMS. Before the analytes enter the IMS, they pass the MCC, which is built of about 1000 capillaries with a diameter of 40 µm. Every capillary is coated with a gel on the inside. The thickness of the gel layer is 200 nm. In particular, the OV-5 phase (5% Phenyl, 95% dimethyl polysiloxane) is used as gel. Pushed through the MCC by carrier gas, the analytes may bind to the gel, depending on their affinity. Thus compounds with higher binding affinity need more time to pass the MCC than others. The analytes that passed the MCC will be piped into the ionization chamber of the IMS. The ion-shutter opens periodically (every 100 ms) and releases the ionized molecules into the drift tube. The time a compound needs to pass the MCC is called its retention time. The time needed to pass the drift tube of the IMS afterwards is referred to as drift time. The sequence of measured IMS spectra is called MCC/IMS measurement. A complete measurement typically takes 10 minutes. 

### 2.2. Data: Measurement and Peak Description 

The measurement we obtain from BioScout is the measured voltage at the Faraday plate over two time axes. To describe the drift time independently of technical properties like drift tube length, drift gas flow or electric field strength, we use a normalized unit, called inverse reduced mobility (Vs/cm^2^). The BioScout yields 12, 500 data points per single IMS spectrum at highest resolution, which equals to a sample rate of 250 kHz. Let *T* be the set of possible x-axis values (inverse reduced mobility or “drift times”) and *R* be the set of possible y-axis values (“retention times”). 

Omitting meta-information, e.g., device adjustment parameters, we obtain an |*R*| × |*T*| matrix *S* =(*S*(*r*,*t*))*_r_*_∈_*_R,t_*_∈_*_T_*, which can be visualized as a heat map (Figure 1). A single IMS spectrum corresponds to a row of the matrix, while a column of the matrix is called contour line. The whole matrix is called IMS chromatogram. 

In each measurement, several regions with high signal values stand out; these regions called peaks are defined by the position and the intensity of the local maxima in this region. These parameters give information about a particular VOC and its concentration. A characteristic high-intensity signal can be found in all spectra of an IMS chromatogram, called reactant ion peak (RIP), see (left side in Figure 1). 

In the literature we may find several mathematical formalizations of what a peak is, e.g., a parametric model describing the shape with statistical functions [11,12]. In this paper, we simply define a peak within one MCC/IMS measurement with three parameters: retention time *r*, inversed reduced mobility *t* and signal intensity *s*. In other words, a peak *P* is a triple *P* = (*r,t,s*). Since we are given with multiple MCC/IMS measurements, we extend this peak description by the measurement index *i* resulting in *P* = (*i,r,t,s*). For convenience, we use the notations *i*(*P*) := *i*, *r*(*P*) := *r*, *t*(*P*) := *t*, *s*(*P*) := *s* to define the projections on each component. 

In this paper, we consider a dataset of 69 measurements, to which we apply the homogenization, filtering and individual preprocessing steps described in the remainder of this section. Note that our data set contains two measurements that are discarded during the homogenization phase, which we describe next. 

### 2.3. Homogenizing and Filtering a Set of Measurements

Given measurements from different sources, different hospitals, for instance, we have to ensure that all measurements are comparable. In our study, we set up a list of rules and restrictions the measurements have to satisfy.

- Drift gas flow: 100 ± 5 mL/min
- Sample gas flow: 100 ± 5 mL/min
- Carrier gas flow: 150 ± 5 mL/min
- MCC temperature: 40 ± 2 °C
- Drift gas: the same value for all measurements in the set
- Polarity: the same value for all measurements in the set

The next step is to determine the measurement with the lowest retention time range, the so-called cut-off time. In each MCC/IMS measurement, the set of measured spectra is reduced to those before the cut-off time. All these rules ensure that the measurements in our set are generally comparable. 

Since most signals occurring at retention times < 5s originate from the MCC/IMS device itself, they were discarded as well.

### 2.4. Preprocessing an MCC/IMS Measurement

We apply four more preprocessing steps to further reduce the noise in the now homogenized data sets, as briefly introduced in the following. The first step is a RIP compensation filter, which subtracts the median of each contour line from each data point. To further de-noise the data, in a second step, a low-pass filter is applied that removes high frequencies from the spectrum. This filter is implemented by a two-dimensional discrete Fourier transform (DFT) [13]. The third step smooths the data by using a two-dimensional Gaussian blur [14]. Finally, a one-dimensional Savitzky–Golay filter smooths every single spectrum computing a weighted average across the drift time axis with a window size of 9 data points [15]. 

## 3. Methods

We evaluate five methods for peak detection: (1) manual peak detection by an expert, which we will refer to as the “gold standard”; (2) an automated local maxima search (LMS); automated peak detection in both (3) IPHEx and (4) VisualNow; and (5) a peak model estimation approach. The input for each method is the output of the preprocessing step described above. All of them transform the given data matrix into a list of potential peaks (*i,r,t,s*) such that we arrive at a list of peaks for each tool for each measurement, *i.e*., each patient. Separately for each tool, the peak lists are merged over all patients such that we are given with a set of peaks and a list telling us whether we observe this peak in a certain measurement (patient) or not. This matrix can then be interpreted as a list of feature vectors that in turn can be used for classification. In the following, we describe the peak detectors and the merging procedure. Afterwards, we detail the evaluation scheme and briefly introduce the used machine learning methods. 

### 3.1. Peak Detection Methods

#### 3.1.1. Manual Peak Detection in VisualNow

The easiest and most intuitive way of peak detection is manual evaluation of a visualization of the measurement. The human eye and visual cortex is optimized for pattern recognition in 3D. Therefore one can immediately spot most of the peaks in the measurement (cf. see Figure 1). The VisualNow software allows to visualize the measurement and to pick regions that define a compound. While analyzing a whole set of measurements, this procedure has to be done for each of them. There are several drawbacks of this procedure. On the one hand, it is time consuming and therefore inappropriate in a high-throughput context; on the other hand, the results depend on a subjective assessment and are therefore hardly reproducible. Nevertheless, it is still the state-of-the-art for the evaluation of smaller MCC/IMS data sets. Thus we are using manually created peak lists as “gold standard” in this paper and evaluate if automatic approaches are generally able to compete. 

#### 3.1.2. Automated Local Maxima Search

This approach identifies local maxima, which is the simplest computer-aided way to identify peaks. Most of the more complex methods, such as the below described, use this as initial procedure to find “seeds” for their algorithms. A point (*r,t*) is a local maximum if all 8 neighbors in the matrix have a lower intensity than the intensity at (*r,t*). In addition, we call the neighborhood of a point (*r,t*) “significant” if its own intensity, that of its 8 neighbors, and that of A additional adjacent points, lie above a given threshold *I*. Here, we use intensity threshold *I* = 10 and *A* = 2 additional points. Our implementation reports all such local maxima that satisfy the “significant” neighborhood condition for these values. 

#### 3.1.3. Automated Peak Detection in VisualNow

VisualNow (B&S Analytik, Dortmund, Germany) is a commercial software package, which is able to visualize and analyze MCC/IMS data. Besides providing visual means for manual peak picking, it also offers automated peak detection. The method consists of two main steps. In the first step, each data point (*r,t*) in a measurement is assigned to one of the two classes, either peak or non-peak, using a clustering method similar to the traditional k-means [16]. In a second step, neighboring data points that belong to the same peak are linked together. This procedure was first introduced for MCC/IMS data by Bader *et al.* ([17]) and called “merging regions algorithm”. Finally, each peak of the analyzed measurement is characterized by the centroid point, *i.e*., that data point in *S*, which has the smallest mean distance to all other points of that were assigned to the peak region [9]. 

#### 3.1.4. Automated Peak Detection in IPHEx

IPHEx is a tool developed by Bunkowski [10] for visualizing, analyzing and managing MCC/IMS data. It mainly builds on the watershed method [18]. The data is treated as a field of hills and valleys. An imaginary water level is continuously lowered while uncovering more and more of the hills. The algorithm starts with labeling the highest data point. For each lower water level, the new uncovered data points inherit the label of their adjacent labeled neighbor. Finally, when all possible labeling actions occurred but some data points are still unlabeled, the highest (un-connected) data points become new peaks and receive new labels. The algorithm runs until all data points are labeled or the level drops below a defined threshold. For more details the reader is referred to [10]. 

#### 3.1.5. Peak Model Estimation

The peak model estimation (PME) was initially designed not as a method to detect peaks but for describing them with statistical mixture models of parametric distributions. At its core it uses an expectation maximization (EM) algorithm, a computational method to optimize the parameters for a mixture model from a given (or “guessed”) set of starting values. Hence, PME depends on initial “seed” results from any peak detection method, *i.e*., an external peak list. Theoretically all previously described methods are suitable. However, PME includes a method described by Fong *et al.* [19], which is based on finding roots in the first derivatives of both spectra and chromatograms. Essentially, the peak list is used to determine the number of models for the PME as well as the start positions for the EM algorithm. Each model function in the mixture describes the shape of one peak by a product of two shifted inverse Gaussian distributions and an additional peak volume parameter. Several of these model functions plus a noise component then describe the whole measurement. Typically the results of peak detection methods are discrete indices. However, this modeling approach uses continuous functions and thus provides a continuous, non-integer peak position (“between” positions *S*), which is presumably more precise than the discrete peak positions provided by other approaches. More details may be found in Kopczynski *et al.* [11]. 

#### 3.1.6. Postprocessing

Given is a list of putative peaks for each measurement for each peak picker. For each peak detector, we now aim to create a matrix of peaks and patients that provides the intensity of the corresponding peak. Implicitly, this matrix describes whether we observe a certain peak in a certain measurement (patient) or not, and if so with which intensity. This matrix can then be interpreted as a list of feature vectors, which can be utilized for subsequent classification (next section). Therefore, we need to (1) “repair” peaks that are too close to each other to be two separate peaks in reality but should be merged to a single one instead. We further need to (2) account for peaks that occur in different measurements at “slightly” different positions, which we will refer to as “peak clusters” in the remainder of this paper. 

For both steps, we utilize the peak merging method described by Boedecker *et al.* [9]. Initially, all peaks are sorted by descending intensity. Potential peaks *P* and *Q* with *s*(*P*) > *s*(*Q*) are merged (*i.e*., labelled *P*) if the following conditions are satisfied: |*t*(*P*) − *t*(*Q*)| < 0.003 and |*r*(*P*) − *r*(*Q*)| < 3.0 + *r*(*P*) · 0.1. Note that this procedure is applied solely to the automated peak detection results. The manual peak detection with VisualNow directly resulted in a list of peak clusters for each measurement. 

### 3.2. Evaluation Methods

After postprocessing, we are given with a set of peak clusters for each peak detection tools as well as the intensity of the corresponding peak cluster in each measurement. 

We assume that one peak cluster originates from one specific compound or metabolite. Under the condition that a certain peak cluster is important for a good classification between the healthy control group and disease patients, we may use further bioanalytic techniques to determine to which compound the peak cluster corresponds. In modern biomarker research, we essentially seek to find correlations between metabolite(s) and a certain disease, thereby suggesting a conditional relationship that a certain disease may provide a certain VOC. Therefore it is desirable that a peak detection method is able to find relevant peaks that trigger a good classification performance. 

To compare the results of the different peak detection methods, two different steps are performed. First, we analyze the overlap of the peak lists and peak clusters to assess their agreement. In a second step we evaluate the classification performance of the different detection methods. 

#### 3.2.1. Peak Position Comparison

Recall that a peak in a measurement is described as *P* = (*i,t,r,s*) (see Section 2.2), where *i* is the measurement index, *t* the inverse reduced mobility, *r* the retention time and *s* the intensity. The different peak detection methods result in different peak cluster positions such that simply studying their intersection is infeasible because two peak clusters that refer to identical molecules would only be recognized as the same if the clusters had exactly the same positions. This is very unlikely. To overcome this problem, we adapted the conditions defined in Section 3.1.6.. Let *V*, *W* be two peak lists generated by two different peak detection methods. Two peaks clusters *P* ∈ *V* and *Q* ∈ *W* are mapped (considered identical), if they fulfill the conditions defined in Section 3.1.6. and if *i*(*P*)= *i*(*Q*). The overlap of list *V* with list *W* is defined as the number of peaks in *V* that can be mapped to at least one peak in *W*. The resulting mapping count table is not symmetric, since each peak of list *V* can be mapped to more than one peak from list *W*. 

#### 3.2.2. Machine Learning and Evaluation

The result of the postprocessing step is a feature matrix, *i.e*., intensities of peak clusters that presumably resemble the abundances of the corresponding molecules for each of the samples (patients). 

In our case the samples are assigned by two classes: *K* for control and *D* for diseased. We use two distinct standardized machine learning methods to get an overview of the potential of the peak detection methods and the different classification strategies. On the one hand we choose a linear learning technique, namely the linear support vector machine. On the other hand a non-linear method is selected (random forest) to evaluate potential non-linear dependencies within the data. 

**Linear Support vector machine:** SVM is one of the most widely used statistical learning methods. This technique is based on the maximization of the margin, defining the region surrounding the hyperplane that best splits the different classes. In 1992, Boser *et al.* suggested the application of the kernel trick as a solution to create non-linear classifiers, for example by using the Gaussian radial basis function; see [20] for more details. SVM was implemented using the *e1071* package [21], with the cost and tolerance parameters of the linear SVM set to 100 and 0.01. 

**Random Forest:** Random forest builds a large collection of de-correlated trees by using bootstrapping. It averages the results (regression) or uses a majority vote (classification). It is based on the bagging strategy, which is a sampling technique applying a method with low-bias and high-variance on subsets of the data. Decision trees are so-called perfect candidate methods. They can capture complex interactions in the data and are unbiased if grown sufficiently deep. To reduce the high-variance of the trees, the outcome is averaged. See Hastie *et al.* [16] for more details. The random forest classification and feature selection are performed using the *randomForest* R package, by Liaw and Wiener in 2002 [22]. Again, standard parameters were used. 

**Evaluation:** In order to achieve a robust estimation of the quality, the data is evaluated in a ten-fold cross validation (CV) environment. In settings with comparably small data set sizes, the normal split into training-, validation-and test-set leads to relatively noisy estimates of the predictive performance. Therefore, we use CV to give an estimate for the actual accuracy of the predictive model. To ensure that each subset covers the variety of both classes, the classes are balanced within each CV subset. Furthermore the CV procedure was repeated 100 times using 100 different ten-fold cross validation sets. Thereby we can analyse the robustness of the different peak sets towards changes in the measurement set. 

The classification results are evaluated based on the feature matrix emerging from the five peak detectors by using different quality measures: (1) accuracy (ACC); (2) the AUC, which is the area under the receiver operating characteristics (ROC) curve [23]; (3) the sensitivity; (4) the specificity; (5) the positive predictive value (PPV); and (6) the negative predictive value (NPV). Furthermore, we give mean and standard deviation of the AUC in boxplots. 

Finally, we investigate whether the feature sets and their model performance are susceptible to classification parameter tuning using a single ten-fold cross validation. We will pick the classifier that performs worse since the potential for improvement will be higher. The parameters are varied systematically. In addition, we randomize the class labels allowing us to judge the robustness to small parameter changes on both the original class labels and the randomized class labels (expected to lead to a decreased classification performance). 

## 4. Results and Discussion

We apply the peak detection methods and evaluation criteria to 67 measurements (cf. Section 2.2). Table 1 gives an overview on the results of the postprocessing. After merging the overlapping peaks of the peak lists, the automatic VisualNow and IPHEx methods show the by far largest number of peaks, between 4000 and 6000. The manual peak picking, local maxima as well as the peak model estimation methods find a similar amount of peaks, “only” about 1500. The number of peak clusters is almost constant over all methods, it varies between 40 and 90. An exception is the automated IPHEx peak picker, which finds 420 clusters. Both VisualNow-based methods, manual as well as automated, find a comparably high number of potential peaks related to a low number of resulting peak clusters. The reason lies with the VisualNow implementation. Once a potential peak was found in one measurement (out of the 67), it automatically “finds” a peak at this position in all other 66 measurements (presumably with low intensities), even if no actual peak exists. This results in the observed high number of peaks, which are mainly noise and, as we will demonstrate later, may lead to problems within the classification procedure. In contrast, the IPHEx, local maxima and PME approaches only assign intensities to peak clusters for those measurements where a peak at the corresponding position is detectable. 

**Table 1:** The number of peaks detected by all methods. The second column gives the number of peak clusters after merging the peak lists (postprocessing).

	# Peaks	# Peak Clusters
Manual VisualNow	1661	41
Local Maxima Search	1477	69
Automatic VisualNow	4292	88
Automatic IPHEX	5697	420
Peak Model Estimation	1358	69

**Table 2:** Overlap of the five peak detection methods. The overlap of the peak list *A* (row) and peak list *B* (column) is defined as the number of peaks in *V* that can be mapped to at least one peak in *W*. Note that the resulting mapping count table is not symmetric.

	Manual	LMS	VisualNow	IPHEx	PME
Manual	1661	911	1522	1184	791
Local Maxima	868	1477	1096	1074	1128
VisualNow	2667	2233	4292	2341	2082
IPHEx	1112	1009	1157	5697	912
PME	737	1086	983	926	1358

### 4.1. Peak Position Comparision

The overlap between the different peak detection methods is summarized in Table 2. The comparison of the peak cluster lists shows a large similarity of both peak lists created with VisualNow, *i.e*., most peaks found in the manual evaluation were also identified with the automatic peak detection of VisualNow. The IPHEx water level approach creates a huge set of peaks. Hence it finds many peaks detected by the other approaches as well (≈ 70% in average). Nevertheless IPHEx seems to be less redundant than the automated VisualNow method since hardly any peaks from the other sets occur several times within the IPHEX set. Local maxima search and the PME method overlap highly (in both directions, ≈ 80%). 

### 4.2. Evaluation by using Statistical Learning

The evaluation of the machine learning performance is shown in Table 3 and Table 4. Table 3 presents the results of the linear support vector machine indicating that all methods perform almost equally well. The manual, local maxima and automatic VisualNow peak detection methods perform worst, in terms of AUC as well as accuracy. The automatic peak detection in IPHEx shows a slightly better AUC and performs best in terms of accuracy 73%. The peak detection method that produced the most informative features for the linear method in terms of AUC ≈ 82% is the peak model estimation approach. 

**Table 3:** Classification Results of the linear support vector machine. The quality measures are the AUC, accuracy (ACC), sensitivity, specificity, positive predictive value (PPV) and negative predictive value (NPV).

	AUC	ACC	Sensitivity	Speciﬁcity	PPV	NPV
Manual VisualNow	77.4	70.9	69.7	72.4	75.7	65.9
Local Maxima Search	77	67.8	70.6	64.4	71	64
Automatic VisualNow	76.6	68.3	66.8	70.1	73.4	63.1
Automatic IPHEx	79.8	73	70.5	76	78.4	67.6
Peak Model Estimation	82.2	72.2	77.2	66.1	73.7	70.1

**Table 4:** Classification Results of the random forest. The quality measures are the AUC, accuracy (ACC), sensitivity, specificity, positive predictive value (PPV) and negative predictive value (NPV).

	AUC	ACC	Sensitivity	Speciﬁcity	PPV	NPV
Manual VisualNow	86.9	76.3	78.7	73.4	78.5	73.6
Local Maxima Search	80.8	70.5	75	64.9	72.5	67.8
Automatic VisualNow	81.1	71.9	75.6	67.3	74.1	69.1
Automatic IPHEx	80	68.9	72.8	64	71.4	65.6
Peak Model Estimation	81.9	74.2	81.6	65	74.2	74.1

Table 4 shows the classification results of the random forest method. Again, all methods vary little in their performance. The best set of features for this machine learning method was generated by the gold standard (Manual VisualNow). The manual detection shows an accuracy of ≈ 76% and an AUC of ≈ 87% and also outperforms all other peak detection methods in all of the quality indices. The peak model estimation performs slightly better in terms of AUC ≈ 82% and accuracy ≈ 74%, as well as most of the other measures. 

**Figure 4:**
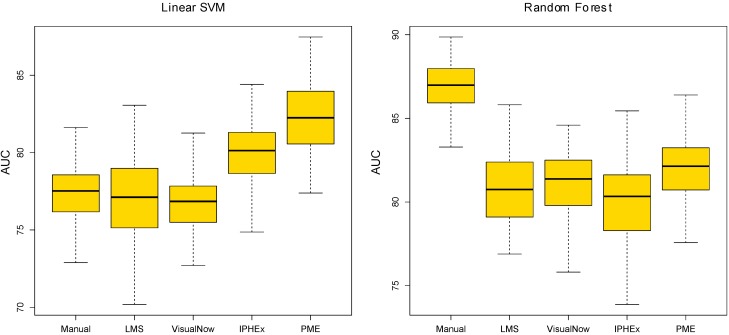
Boxplots of 100 runs of the ten-fold cross validation for both, the linear SVM and the random forest method.

**Data Robustness:** Figure 4 shows boxplots of the list of AUCs generated by 100 runs of the ten-fold cross validation. The prediction results of the linear SVM with the manual and automated VisualNow methods are the most stable, while the local maxima search shows the highest variation. The PME approach has a reasonable robustness and performs better than the simple methods in almost all runs. In comparison, the AUC-measured classification performance with random forest is most robust for the gold standard and the PME approach. The other automated methods introduce larger variations, in particular IPHEx. 

**Figure 5:**
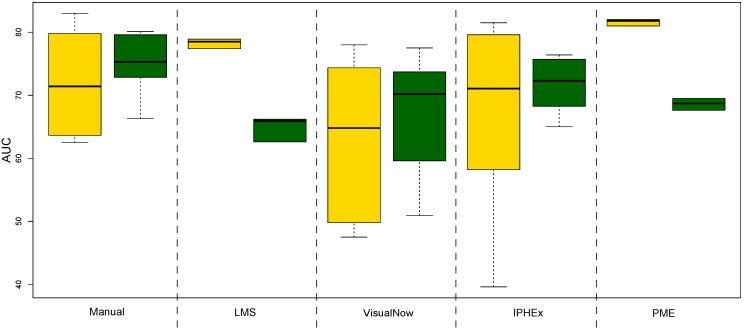
Boxplots illustrating the variation within the linear SVM tuning results in a single ten-fold cross validation run. The yellow boxes show the results when tuning the original feature sets. The green boxes show the results when tuning the randomly labeled feature sets.

**Tuning Robustness:** Finally we investigate if the feature sets and their model performance are susceptible to parameter tuning for the worse performing classifier: the linear SVM. Therefore, we systematically vary the cost and tolerance parameters ({0.1,1.0,100,1000} and {0.01,0.1,1}, respectively) and in a second run we randomize the class labels. The result of this analysis is shown in Figure 5, which plots the variance of the AUC for both the original labels (left) as well as the randomized labels (right). The results of the robustness analysis of random forest is shown in the Appendix Figure A1. 

At first glance, Figure 5 indicates that the performance (AUC) of the manual and automated VisualNow, as well as the IPHEx peak detection feature set, can be heavily improved by tuning the classifiers’ parameter sets. However, when considering the results for the randomized labels, these three tools seem to generate peak clusters that are prone to overfitting, most likely resulting from the high number of detected potential peaks. We would generally expect to observe a drastic drop in the classification quality for the randomized labels compared with the real labels, which is not clearly observed for all methods (overfitting), but LMS and PME. In addition to its comparably low susceptibility to overfitting, PME has a quite small variability in AUC, indicating stable classification results. In contrast to the results of the linear SVM, the random forest tuning results show that this method is considerably less stable. 

In contrast to the tuning results of the linear SVM, most data sets show considerable smaller potential for tuning of the random forest. Furthermore, for all data sets, we observe a drastic drop in classification quality for the randomized labels, compared with the original labels. One can generally say that random forest classification appears to be more robust in terms of overfitting on this dataset. 

## 5. Conclusions

To summarize, we compared four different approaches for automated peak detection on medical MCC/IMS measurements: local maxima search, automated VisualNow, IPHEx watershed transfor­mation, and peak model estimation in comparison with the gold standard, manual peak picking. In particular, we investigated their impact on the goodness of classical machine learning approaches that are used for separating patients into “healthy” and “not healthy”. This is crucial in current biomarker research since it influences medical decision making. 

Our results indicate that the automated approaches can generally compete with manual peak picking protocols carried out by experts in the field, at least with regards to subsequent health status classification. Although the manual peak picking remains the gold standard, in medical studies yielding huge amounts of data, one has to weigh the tradeoff between a slightly higher accuracy (manual) and a huge increase in processing speed (automatic). For example, a set of 100 MCC/IMS measurements is processed in less than ten minutes, whereas an expert would need more than ten hours. However, automated peak detection methods would process every kind of input, whereas a domain expert would immediately recognise erroneous data. Nevertheless, the quite recent peak model estimation approach PME slightly outperforms the other automated methods though all methods perform almost equally well. PME is also most robust against overfitting. 

We conclude that all current automatic peak picking methods provide generally good results. They are quite sensitive but might be improved in their specificity by reducing the number of peaks they predict per measurement. This will, however, be difficult to implement while keeping the comparably high sensitivity rates. In the future, we will use the introduced pipeline for more and, in particular, larger data sets. Data and additional material can be found at [24]. 

## Figures and Tables

**Figure A1:**
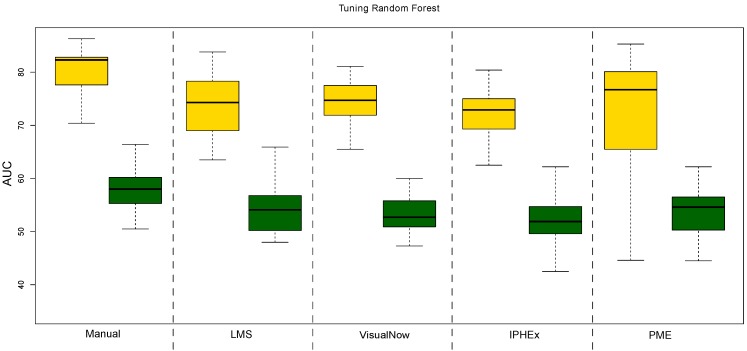
Boxplots illustrating the variation within the random forest tuning results in a single ten-fold cross validation run. The yellow boxes show the results when tuning the original feature sets. The green boxes show the results when tuning the randomly labeled feature sets.
